# Effects of rotation corn on potato yield, quality, and soil microbial communities

**DOI:** 10.3389/fmicb.2025.1493333

**Published:** 2025-04-16

**Authors:** Zhicheng Zhang, Jiying Sun, Dan Wang, Tuanrong Lin, Yuhe Yin, Wei Wang, Yufeng Wang, Zhen Wang, Longqiu Fan, Xinlei Jiao

**Affiliations:** ^1^Inner Mongolia Agricultural University, College of Agriculture, Hohhot, China; ^2^Ulanqab Institute of Agricultural and Forestry Sciences, Potato Research Laboratory, Ulanqab, China; ^3^Jining Normal University, College of Life Science and Technology, Ulanqab, China

**Keywords:** potato, maize, continuous cropping, soil microbial, metagenome

## Abstract

**Introduction:**

Potato is an important crop that can be used both as grain and vegetable in northern China. However, the continuous cropping system of potato has led to a sharp decline in its yield and quality. As one of the effective strategies to alleviate the continuous cropping obstacle, crop rotation has received extensive attention in agricultural practices. On this basis, we have conducted an in-depth exploration of the effects of the potato-maize rotation system on the structure and diversity of the soil microbial community, aiming to analyze the internal correlation mechanism between the structure of the soil microbial community and the yield and quality of crops.

**Methods:**

This study was based on fields that had been under potato monoculture for five years and established six experimental treatments: potato-potato-potato (IR-A), potato-maize-potato (IR-B), potato-maize-maize (IR-C), potato-potato-potato (RF-A), potato-maize-potato (RF-B), and potato-maize-maize (RF-C).

**Results:**

The results showed that under the IR planting model, IR-B significantly increased potato yield and vitamin C content while reducing reducing sugar content compared to IR-A (*p* < 0.05). In the RF planting model, RF-B significantly increased potato yield, starch content, and vitamin C content compared to RF-A (*p* < 0.05). Microbial community structure results indicated that crop rotation significantly enhanced the relative abundance of microorganisms such as *Bradyrhizobium*, *Pseudomonas*, *Sphingomonas*, *Purpureocillium*, *Streptomyces*, and *Halovivax* (*p* < 0.05). These microorganisms are involved in the cycling of carbon, phosphorus, and other nutrients in the soil, playing an important role in promoting root growth, organic matter decomposition, and alleviating soil salinization. The LEfSe and RDA indicated significant differences in microbial communities between monoculture and crop rotation (*p* < 0.05), with soil slow-growing rhizobia, Burkholderia, and actinomycetes positively correlated with potato yield and quality. Additionally, KEGG functional annotation of different treatments revealed that K00239, K00626, K01681, and K01915 were involved in three key metabolic pathways related to carbon and nitrogen. A total of 20 significantly enriched pathways were identified (*p* < 0.05), among which K01681 is involved in the tricarboxylic acid cycle and is a differential gene in the RF-B treatment, suggesting that the efficient expression of K01681 during crop rotation contributes to the material cycling of the soil ecosystem. LEfSe analysis of the bins revealed that under the RF-C treatment, the relative abundance of Hyphomicrobiales was significantly higher than in other treatments (*p* < 0.05). Hyphomicrobiales are involved in the nitrogen fixation process and play an important role in soil nutrient cycling and plant nutrition. In summary, the potato-maize rotation significantly altered the composition of soil microbial communities (*p* < 0.05), increasing the relative abundance of beneficial microorganisms. This change helps maintain the health of the soil ecosystem, promotes nutrient cycling, reduces the incidence of diseases, and effectively improves both the yield and quality of potatoes.

**Discussion:**

The potato-maize rotation significantly altered the composition of soil microbial communities (*p* < 0.05), increasing the relative abundance of beneficial microorganisms. This change helps maintain the health of the soil ecosystem, promotes nutrient cycling, reduces the incidence of diseases, and effectively improves both the yield and quality of potatoes.

## Introduction

As a vital biological indicator of soil ecosystems, soil microbial diversity not only reflects the health status of the soil but also has a direct impact on soil fertility, nutrient cycling, and plant growth (Xiao et al., 2024). With the advancement of sustainable agricultural development, the implementation of crop rotation to alter crop planting systems can significantly change the structure and function of soil microbial communities, thereby profoundly influencing soil health and crop productivity (Tan et al., 2022). In crop rotation systems, the alternating cultivation of potato and maize demonstrates potential advantages in enhancing soil fertility and crop yield (Li et al., 2023). As two key food crops, potato and maize exhibit distinct root structures and nutrient demands. Potatoes have shallow roots primarily distributed in the upper soil layers, while maize roots are deeper and wider, extending into the middle and lower soil layers. This difference in root distribution enables the rotation system to utilize soil nutrients and water resources more efficiently, reduce competition for resources, and provide a richer source of organic matter for soil microbial communities.

Currently, it has been reported that continuous cropping systems adversely affect soil microbial community structure and soil quality, resulting in decreased crop yields across various crops, including potato (Du et al., 2023), sweet potato (Pang et al., 2021), tomato (Wang et al., 2018), and soybean (Liu H. et al., 2019). Studies have shown that crop rotation can significantly alter the diversity of soil microbial communities, and these microbial changes directly influence the absorption and transformation of soil nutrients (He et al., 2024). Wang utilized Illumina MiSeq high-throughput sequencing technology to study the changes in soil community structure and function under traditional planting patterns. Compared to continuous cropping of potatoes, the relative abundance of *Monographella* increased, while the relative abundance of *Humicola* decreased in the rape-wheat-beet-potato (Bn-Ta-Bv-St) and wheat-rape-barley-potato (Ta-Bn-Hv-St) models. In the potato-wheat-beet-potato (St-Ta-Bv-St) treatment, only the relative abundance of *Colletotrichum* increased. The relative abundance of *Mortierella* increased solely in the wheat-beet-wheat-potato (Ta-Bv-Ta-St) treatment (Wang et al., 2024).

Regarding specific changes in the microbial community, the bacterial and fungal community structures in soil under rotation conditions show significant differentiation. Research on the effects of continuous cropping and potato-tartary buckwheat rotation on soil community structure in Yunnan indicated that continuous cropping increased the abundance of Mortierellomycota, Ascomycota, and *Mortierella*, whereas rotation decreased the abundance of Ascomycota and *Plectosphaerella* in the soil (Du et al., 2023). Wang analyzed the effects of legume and potato rotation on rhizosphere soil, finding that, compared to continuous cropping, the number of bacteria and actinomycetes in the potato-faba bean-potato treatment increased by 38.18 and 52.64%, respectively, while the number of fungi decreased by 18.1% (Wang Y. et al., 2023). The relative abundance of Proteobacteria, Actinobacteria, and Acidobacteria in the soil of continuously cropped potatoes was higher, while the relative abundance of Ascomycota in soil fungi was lower. Additionally, the number of *Spizellomyces* was higher in fallow and rotation soils, indicating that long-term continuous cropping of potatoes reduces soil microbial diversity, alters dominant microbial populations, and results in an unbalanced soil microbial community structure (Tan et al., 2022). Moreover, crop rotation can enhance the stability of soil ecosystems by increasing the complexity of microbial communities. Under continuous cropping conditions for potatoes, the microbial genera (*Pseudomonas*, *Colletotrichum*, *Plectosphaerella*, *Fusarium*, and *Verticillium*) associated with disease infection increased under the oat-faba bean-potato-oat rotation system. Conversely, the relative abundance of microbial genera (*Devosia*, *Aeromicrobium*, *Paraphoma*, and *Papiliotrema*) linked to organic residue degradation, soil nitrogen cycling, and disease inhibition was significantly increased (Qin et al., 2022). Additionally, crop rotation systems can indirectly influence the structure of microbial communities by altering the physical and chemical properties of the soil.

Inner Mongolia is recognized as one of China’s principal potato-producing regions (Jin et al., 2018). In recent years, challenges such as reduced potato yields and the prevalence of soil-borne diseases stemming from continuous cropping have emerged, necessitating urgent solutions (Tan et al., 2022). Maize, a gramineous crop, serves as a significant food source in Inner Mongolia and presents an ideal candidate for potato rotation. The practice of potato-maize rotation is common in northern China; however, the underlying mechanisms by which this rotation alleviates the obstacles associated with continuous potato cropping remain unclear. In this study, we established a 5-year continuous cropping system for potatoes and selected the main local maize variety “Xianyu 1219” known for its drought resistance, cold tolerance, and high yield, to rotate with the potato variety “Qingshu 9.” We employed Illumina MiSeq high-throughput sequencing technology to investigate the soil microbial communities associated with both potato rotation and continuous cropping under various cropping systems. Additionally, we examined the potential impacts of maize rotation on potato growth and the soil environment, aiming to elucidate the intrinsic relationship between soil microbial community structure and both yield and quality across different cropping systems. The findings of this study will provide a theoretical foundation and technical support for addressing the challenges posed by continuous potato cropping.

## Materials and methods

### Experimental materials and design

The main potato variety “Qingshu 9” and maize variety “Xianyu 1219” in northern China were selected as experimental materials. The potato variety “Qingshu 9” was introduced from the Qinghai Academy of Agricultural Sciences and is being bred by the Potato Research Team at the Ulanqab City Agricultural and Forestry Science Research Institute. “Qingshu 9” exhibits excellent performance in terms of yield, quality, and resistance, making it one of the main promotion varieties in the Inner Mongolia region. While “Xianyu 1219” was obtained from Tieling Pioneer Seed Research Co., Ltd. The planting density for potatoes was set at 3.7 × 10^4^ plantsn p^–1^, with a row spacing of 0.90 m and a plant spacing of 0.30 m, resulting in a plot area of 108 m^2^ (30 m × 3.6 m). In contrast, the planting density for maize was 7.5 × 10^6^ plants. I^–1^, with a row spacing of 0.50 m and a plant spacing of 0.25 m, leading to a plot area of 120 m^2^ (30 m × 4 m). The potato seed usage is 2,000-2,500 kg/ha, and the maize seed usage is 300-400 kg/ha. The special compound fertilizer (N-P_2_O_5_-K_2_O:12-19-16) 100 kg/667 m^2^ was applied to the base fertilizer of the potato plot, 10 kg of urea was applied twice at the seedling stage, and 20 kg of high-phosphorus and high-potassium (N-P_2_O_5_-K_2_O:10-20-30) liquid fertilizer was applied in the middle and late stages, which was applied 3-4 times, and the field irrigation was 6-8 times during the whole growth period. The compound fertilizer (N-P_2_O_5_-K_2_O: 15-15-15) 20 kg/667 m^2^ was used as the base fertilizer in the corn field, and the urea 15-20 kg was applied during the growth period, which was applied in 3-4 times, and the field irrigation was 2-4 times during the whole growth period. No irrigation and topdressing in the later stage under rain-fed mode.

Based on the potato field established in 2017, continuous potato planting was conducted for five years, from 2017 to 2021. In the spring of 2022, a portion of the potato fields was rotated with maize, while the remaining plots continued to be planted with potatoes. The potato-maize rotation experiment was carried out from April to October in both 2022 and 2023. Utilizing a randomized block design, a total of six treatments were established, including irrigation (IR) and rain-fed (RF) conditions (Table 1). Each treatment was replicated three times. To minimize inter-treatment interference and the marginal effect, a spacing of 40 cm was maintained around each plot, and protective rows measuring 100 cm were left around the plots.

**TABLE 1:** Experimental design.

Planting pattern	Treatment	Continuous/Rotation mode		
		**2017-2021**	**2022**	**2023**
Irrigation	IR-A	Potato	Potato	Potato
	IR-B	Potato	Maize	Potato
	IR-C	Potato	Maize	Maize
Rain-fed	RF-A	Potato	Potato	Potato
	RF-B	Potato	Maize	Potato
	RF-C	Potato	Maize	Maize

### Description of the experimental site

The experiment was conducted at the experimental base of the Institute of Agricultural and Forestry Sciences in Ulanqab from 2017 to 2023 (40°93′ N, 113°16′ E, 1,396 m). This region experiences a semi-arid continental monsoon climate typical of the middle temperate zone. The annual sunshine duration ranges from 2,850 to 3,250 h, while the average annual precipitation is between 250 and 350 mm. Rainfall is predominantly concentrated from June to August, accounting for approximately 70% of the annual total. Notably, rainfall and temperature increase coincide in this season. The test ground is flat with medium and uniform fertility. Due to the prevailing climatic conditions, only one crop can be cultivated each year.

### Collection and processing of soil samples

**FIGURE 1:**
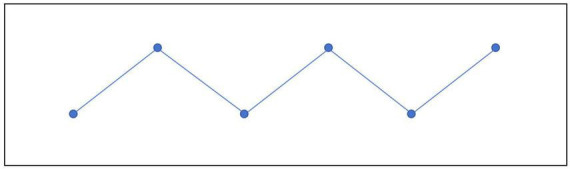
“S” random sampling method.

In this experiment, soil samples were collected and processed using the “S” random sampling method, which is particularly suitable for rectangular plots. During the sampling process, starting from one side of the plot, the sampler moved in a serpentine manner across the plot, selecting sampling points along the route. A total of six sampling points were chosen for each plot. Soil samples were then collected using a soil drill or shovel, ensuring that the depth of collection remained consistent across all points. The collected soil samples were thoroughly mixed to create composite samples (Figure 1; Zhang M. Y. et al., 2020).

The specific methods employed for soil sampling were as follows: Initially, the 0-5 cm topsoil was removed, and samples from the surface soil layer (5-25 cm) were collected. Efforts were made to maintain consistency in both the collection depth and quality across all sampling points. The collected soil was placed in a soil tray, where impurities were carefully removed and the soil was mixed thoroughly. Excess soil was eliminated using the quartering method until the desired quantity was achieved. The final soil sample was then transferred into a sterile bag, labeled appropriately, and placed in an ice box for transport back to the laboratory for further analysis. In the laboratory, the fresh soil samples that had passed through a 2 mm sieve were divided into three portions: one portion was designated for the determination of the physical and chemical properties of the soil, another was allocated for assessing soil enzyme activity, and the third portion was reserved for metagenomic analysis.

### Determination of quality traits

Seven to ten days post-harvest, five tubers weighing approximately 150-200 g were randomly selected from each plot and transported to the laboratory for analysis. The following parameters of the tubers were measured using a spectrophotometer: dry matter, starch, protein, reducing sugar, and vitamin C (Vc). The starch content was determined using the iodine-potassium iodide colorimetric method (Yuan and Zhang, 2009), while the reducing sugar content was assessed through 3,5-dinitrosalicylic acid (DNS) colorimetry (Wayumba et al., 2019). The protein content was quantified using the biuret method (Zhang and Tian, 2007), and the vitamin C content was determined via 2,6-dichloroindophenol titration (Zhang et al., 2007). For the determination of dry matter content, approximately 150 g of potato chips were cut into strips, wrapped in newspaper, and placed in an oven. The samples were subjected to a pretreatment at 105°C for 30 min to kill them, followed by drying at 60°C for 72 h, after which they were weighed. The dry matter content was calculated using the following formula: Dry matter content (%) = (weight after drying/fresh weight) × 100% (Zhao et al., 2017).

### Soil DNA extraction and Illumina sequencing

Soil DNA was extracted using a soil DNA extraction kit provided by Genepioneer Biotechnologies (China). The concentration and purity of the extracted DNA were assessed using a spectrophotometer (Tanon, China) and evaluated via 1% agarose gel electrophoresis, respectively. For DNA fragmentation, the Covaris M220 system was employed, allowing for the selection of fragments approximately 300 bp in length. Library construction was carried out using the VAHTS Universal DNA Library Prep Kit for Illumina V3 (Illumina, United States) (Qin et al., 2010). A total of 18 soil samples were collected for metagenomic sequencing, which was conducted on the Illumina NovaSeq 6000 platform. This process generated a substantial dataset, yielding 141,412,057,534 high-quality reads, with an average sequencing depth of approximately 7.86 Gb per sample.

### Metagenomic sequencing

Fastp v0.20.0 was used to cut the quality of the adapter sequences at the 3′ and 5′ ends of the reads (Chen et al., 2018). Using MEGAHIT v1.2.9, each sample was assembled individually with the parameters set as follows: –min-contig-len 500, –presets meta-large, to obtain the contig sequences for each sample (Li et al., 2015). Then, MetaGeneMark v3.38 was used to predict the open reading frames (ORFs) for the contigs assembled from each sample (length ≥ 500 bp), and the predictions with lengths less than 100 nt were filtered out (Noguchi et al., 2006). CD-HIT 4.6.1 was used to remove the redundancy of ORF prediction results to obtain non-redundant initial Unigenes (Li and Godzik, 2006). The clean data of each sample was compared with the non-redundant initial Unigenes using bwa 0.7.17, and the number of reads on the comparison of genes in each sample was calculated (Li et al., 2008). Kraken 2.1.2 was used to classify Unigenes, and Unigenes were compared with Bacteria, Fungi and Archaea sequences that the abundance information and gene number table of each sample at each classification level were obtained (Wood and Salzberg, 2014). KRONA was used to analyze the relative abundance. Non-parametric factorial Kruskal-Wallis (KW) sum-rank test (nonparametric factorial Kruskal-Wallis rank sum test) was used to detect the characteristics of significant abundance differences, and the groups with significant differences in abundance were found (Ondov et al., 2011). Lefse 1.0 was used to estimate the effect of each component abundance on the difference effect using linear discriminant analysis (LDA) (*p*-value < 0.05, LDA score > 3) (Segata et al., 2011). DIAMOND v2.0.6 was used to compare Unigenes with KEGG functional database for functional classification and pathway analysis. The parameters were set as: blastp, *e*-value ≤ 1e^–5^ (Kanehisa and Goto, 2000).

### Statistical analysis

The DPS 9.01 software was utilized to analyze the differences in yield and quality among various treatments (Zhang et al., 2008). The mean values of the different treatments were compared using the Least Significant Difference (LSD) method at a significance level of 5%, with the data expressed as “mean ± standard deviation.” Using QIIME, various taxonomic richness tables were generated, and alpha diversity distance calculations were performed. Based on the abundance tables at various taxonomic levels, microbial alpha diversity was assessed by calculating the Shannon index and Simpson diversity index to estimate the diversity of microbial communities in the samples (Xiao et al., 2021). A Bray-Curtis distance matrix was constructed based on species abundance tables at various classification levels. Principal Coordinate Analysis (PCoA) was conducted using R version 4.4.0 to assess the differences in species composition associated with different planting patterns and to evaluate the degree of differentiation in soil microbial community structure (Lever et al., 2017). Additionally, redundancy analysis based on a linear model was performed using Canoco 5.15 software to investigate the relationships between yield, quality, and microbial communities (Ter Braak and Šmilauer, 2012).

## Results

### Analysis of potato quality and yield data

The results indicate notable differences in potato yield and quality between rotation and continuous cropping systems (Table 2). Under the irrigation (IR) mode, the vitamin C content of potatoes in the IR-B treatment significantly increased (*p* < 0.05), while the content of reducing sugars significantly decreased (*p* < 0.05). However, no significant differences were observed in protein and starch content (*p* > 0.05). Under the rain-fed (RF) mode, the vitamin C and starch content in the RF-B treatment were significantly elevated (*p* < 0.05). This enhancement may be attributed to the effects of drought stress on the carbon metabolism of potatoes, leading to a significant increase in starch content and reflecting abnormal changes in carbon metabolism. Notably, the tuber yields for IR-B and RF-B treatments increased by 12.4 and 10.4%, respectively. These findings suggest that potato-maize rotation can effectively enhance both yield and quality of potatoes, with the IR mode demonstrating significantly higher yields compared to the RF mode (*p* < 0.05).

**TABLE 2:** Changes of potato yield and quality under different planting patterns.

Treatment	Yield (t/ha)	Protein (%)	Starch (%)	Reducing sugar (%)	Vitamin c (mg/100 g)
IR-A	53.53 ± 0.54 b	3.67 ± 0.12a	14.29 ± 0.20 b	0.29 ± 0.01 a	22.36 ± 0.40 bc
IR-B	61.10 ± 0.75 a	3.81 ± 0.16 a	14.10 ± 0.31 b	0.24 ± 0.01 b	24.04 ± 0.19 a
RF-A	24.02 ± 0.76 d	2.94 ± 0.10 b	14.81 ± 0.31 b	0.29 ± 0.01 a	21.46 ± 0.58 c
RF-B	26.81 ± 0.18 c	3.05 ± 0.05 b	16.11 ± 0.34 a	0.28 ± 0.01 a	20.57 ± 0.35 b

Lowercase letters within a column mean significant differences among treatments (*p* < 0.05), and *p < 0.05 means significant differences of the same treatment between the two time points. Data represent mean values ± SE (*n* = 3).

### Effects of different crop rotation systems on alpha diversity of soil microbial community

**FIGURE 2:**
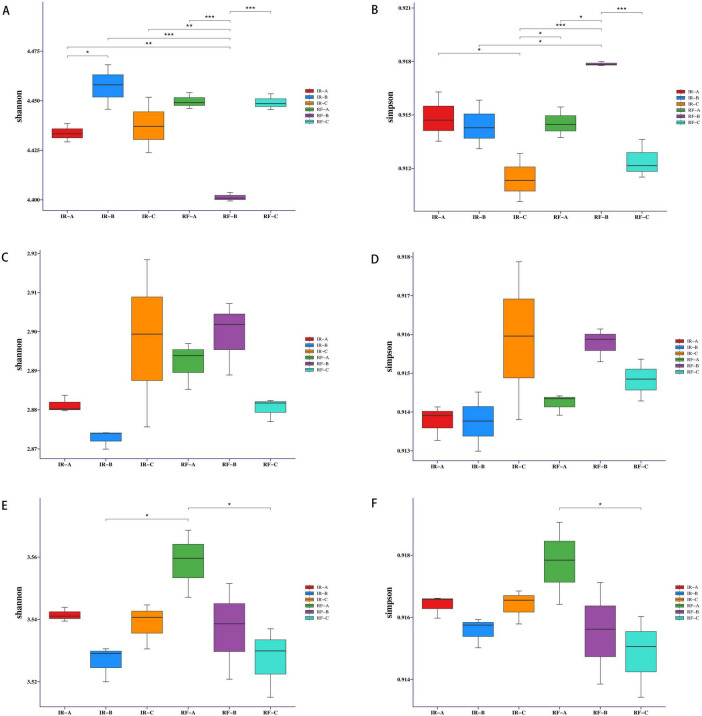
Effects of different treatments on the alpha diversity of bacteria, fungi and archaea. **(A,B)** Denote Shannon indices and Simpson indices for bacteria; **(C,D)** denote Shannon indices and Simpson indices for fungi; **(E,F)** denote Shannon indices and Simpson indices for archaea. *p < 0.05 indicated that there was a significant difference between the two treatments, **p < 0.01 and *p < 0.001 indicated that there was very significant difference between the two treatments.

The alpha diversity index analysis of rhizosphere soil samples revealed significant differences in the Shannon index of soil bacteria between the IR-A and IR-B treatments (*p* < 0.05). This finding indicates that potato-maize rotation can markedly enhance the Shannon index of soil microorganisms, leading to improved microbial richness and evenness. Conversely, the Shannon index for the RF-B treatment was significantly lower than that of the other treatments (*p* < 0.01 or *p* < 0.001) (Figure 2A and Supplementary Table 1). This decrease may be attributed to the excessive depletion of soil moisture and the slow nutrient cycling associated with dry conditions, which hinder the full benefits of the 1-year rotation cycle. Additionally, significant differences were observed in the Simpson index of soil bacteria between RF-B and both IR-B and RF-A treatments (*p* < 0.05), while the difference between RF-B and IR-C as well as RF-C treatments was extremely significant (*p* < 0.001). These results suggest that the dominant species within the RF-B treatment community were more pronounced (Figure 2B and Supplementary Table 1).

The analysis of the Shannon and Simpson indices for soil fungi indicated no significant differences among the various treatments (*p* > 0.05) (Figures 2C,D and Supplementary Table 1).

A significant difference was noted in the Shannon and Simpson indices of soil archaea between the RF-A and RF-C treatments (*p* < 0.05) (refer to Figures 2E,F and Supplementary Table 1). This suggests that rotation under dry farming conditions negatively impacted the richness and evenness of soil archaea. The observed decline in the Shannon and Simpson indices of archaea may be attributed to their limited adaptability to varying soil moisture levels. Following the potato-maize rotation, changes in soil nutrients, moisture, and temperature may have created an environment that was challenging for archaeal communities to adapt to and proliferate within a short timeframe. Consequently, this resulted in a reduction in both the Shannon and Simpson indices for archaea. Conversely, under irrigation conditions, no significant differences were observed in the Shannon and Simpson indices among the treatments (*p* > 0.05).

### Effects of different crop rotation systems on β diversity of soil microbial communities

**FIGURE 3:**
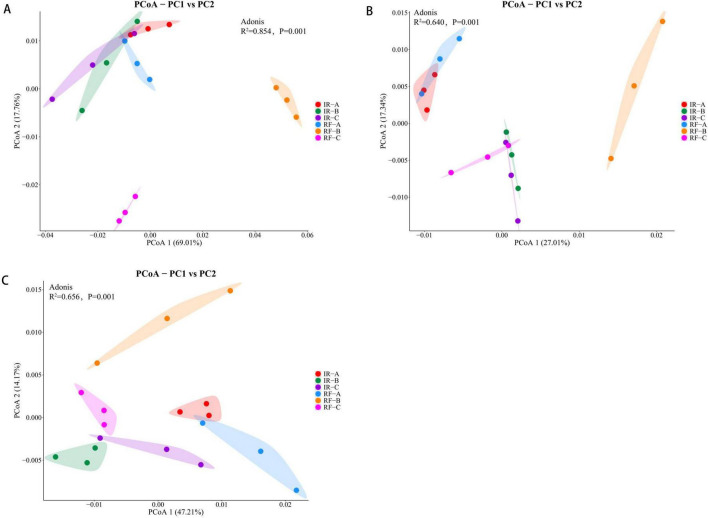
Principal coordinate analysis of soil bacteria, fungi, and archaea under different treatments. **(A)** Represents bacteria, **(B)** represents fungi, and **(C)** represents archaea. Under different treatments, each sample group showed a 95% confidence ellipse.

The principal coordinate analysis (PCoA) of soil microbial communities across different treatments demonstrated that PCo1 and PCo2 accounted for 69.01 and 17.76% of the variance in soil bacterial community structure, respectively. The treatments IR-A, IR-B, IR-C, and RF-A clustered closely together, while RF-B and RF-C were distinctly separated from the others. An analysis of similarity (ANOSIM) further confirmed significant differences in soil bacterial community composition across the various treatments (*R*^2^ = 0.854, *P* = 0.001). This indicates a marked change in the rhizosphere soil microbial community following maize crop rotation under rain-fed conditions (Figure 3A). For the soil fungal community, PCo1 and PCo2 explained 27.01 and 17.34% of the variance, respectively. The treatments IR-A and RF-A showed a relatively concentrated distribution, while IR-B, IR-C, and RF-C formed another cluster, suggesting significant differences in the rhizosphere soil fungal community between continuous cropping and rotation systems (*R*^2^ = 0.640, *P* = 0.001) (Figure 3B). Regarding the soil archaea community, PCo1 and PCo2 accounted for 47.21 and 14.17% of the variance, respectively. The analysis revealed significant differences in the soil archaea community between continuous cropping and rotation cropping systems (*R*^2^ = 0.656, *P* = 0.001). Notably, the observed differences across treatments were largely consistent with those seen in the soil fungal communities (Figure 3C).

### Analysis of genus-level composition of microbial community

**FIGURE 4:**
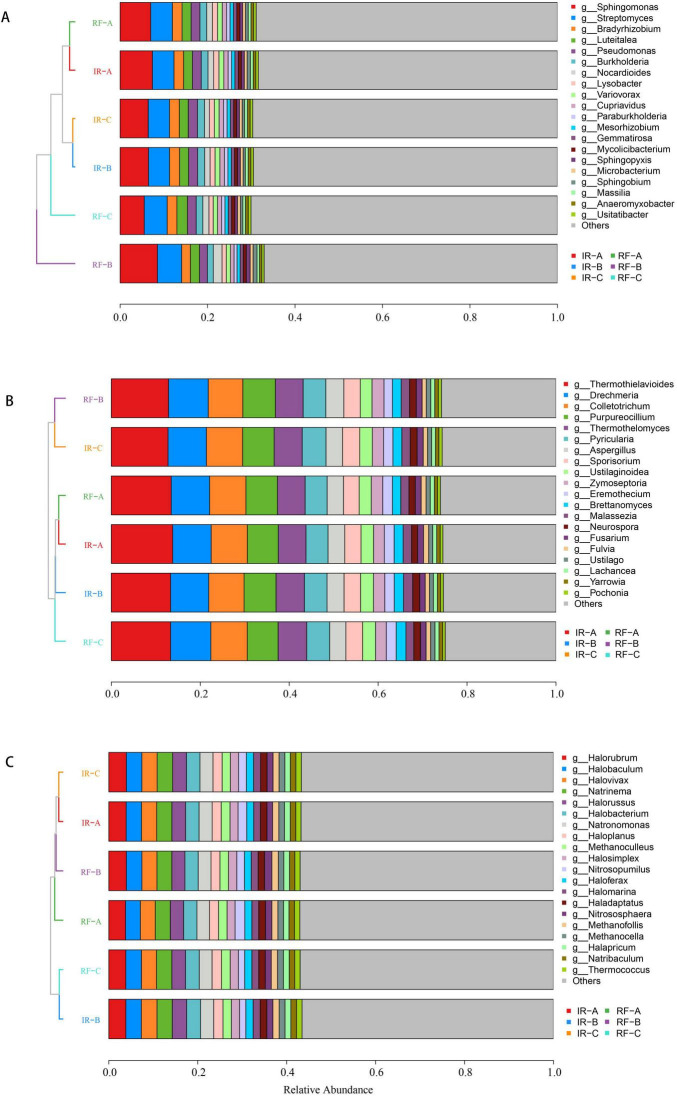
Composition of soil microbial communities of genus level under different systems. **(A-C)** Represent the relative abundance of bacteria, fungi and archaea at the genus levels. The vertical axis is the sample cluster tree, and the horizontal axis is the relative abundance. The color corresponds to the name of different species, and the length of the color block represents the relative abundance of the species represented by the color block.

The analysis of microbial communities at the genus level, focusing on the top 20 species, revealed distinct compositions and clustering patterns among bacteria, fungi, and archaea across the different treatments (Figure 4). In terms of bacterial communities, the six treatments clustered into two main categories, with RF-B forming a separate cluster. The bacterial community composition of the other five treatments exhibited a high degree of similarity among themselves. For the fungal communities, RF-B and IR-C displayed comparable community compositions, which set them apart from the other four treatments. This indicates a notable divergence in fungal community structure. Regarding archaeal communities, RF-C and IR-B showed similar compositions, while some differences were observed when compared to the other four treatments.

The species annotation results revealed that the top five dominant bacterial categories were *Sphingomonas*, *Streptomyces*, *Bradyrhizobium*, *Luteitalea*, and *Pseudomonas* (Figure 4A). Under the Irrigated (IR) system, the relative abundances of *Bradyrhizobium* and *Pseudomonas* in the rotation treatment were significantly higher than those observed in continuous cropping (*p* < 0.05). Additionally, within the IR system, the relative abundance of *Streptomyces* in treatment IR-A was significantly greater than that in IR-B (*p* < 0.05). In the Rain-fed (RF) system, RF-B exhibited the highest relative abundance of *Sphingomonas* (*p* < 0.05). Furthermore, the relative abundance of *Streptomyces* in RF-B was significantly higher than that in RF-A (*p* < 0.05), although no significant difference was detected when compared to RF-C (*p* > 0.05). The relative abundance of *Luteitalea* peaked in RF-C (*p* < 0.05), while *Bradyrhizobium* and *Pseudomonas* were significantly more abundant in RF-A compared to RF-B (*p* < 0.05) (Supplementary Table 2).

The analysis of fungal communities revealed that the top five fungal phyla identified were *Thermothielavioides*, *Drechmeria*, *Colletotrichum*, *Purpureocillium*, and *Thermothelomyces* (Figure 4B). Under the Irrigated (IR) system, the relative abundance of *Thermothielavioides* was highest in treatment IR-A (*p* < 0.05). Conversely, the relative abundance of *Purpureocillium* in IR-B was significantly greater than that in IR-A (*p* < 0.05). In the Rain-fed (RF) system, the relative abundance of *Drechmeria* in RF-C was significantly higher than that in RF-A (*p* < 0.05). Additionally, RF-C exhibited significantly greater relative abundances of *Thermothielavioides* and *Colletotrichum* compared to RF-B (*p* < 0.05). However, no significant difference was found between RF-C and RF-A for these two phyla (*p* > 0.05) (Supplementary Table 2).

*Halorubrum, Halobaculum, Halovivax, Natrinema*, and *Halorussus* were the top 5 archaeal dominant phyla (Figure 4C). Under IR system, the relative abundance of *Halovivax* and *Halorussus* was the highest in IR-B (*p* < 0.05). Under the RF cropping system, the relative abundance of *Halorubrum* was the highest in RF-B (*p* < 0.05), and the relative abundance of *Halorussus* was the highest in RF-C (*p* < 0.05) (Supplementary Table 2).

The conclusions drawn above indicate that crop rotation can significantly enhance the relative abundance of dominant bacterial, fungal, and archaeal communities in the soil’s rhizosphere (*p* < 0.05). These dominant bacteria play a crucial role in various metabolic activities vital for crop life. For instance, genera such as *Bradyrhizobium*, *Pseudomonas*, and *Sphingomonas* are capable of producing growth hormones like auxin and cytokinins, which promote the growth and development of plant roots, thereby improving the plants’ ability to absorb nutrients and water. Moreover, *Pseudomonas* has the unique ability to convert insoluble phosphorus in the soil into forms that are readily available for plant uptake. This process enhances phosphorus absorption by crops, thereby promoting their overall growth. Additionally, microorganisms such as *Purpureocillium*, *Streptomyces*, *Luteitalea*, and *Drechmeria* contribute to the decomposition of organic matter in the soil, including plant residues and cellulose. They break these down into simpler organic compounds and inorganic nutrients, release carbon dioxide, and participate in the soil’s carbon cycle, thus improving nutrient availability and providing more absorbable nutrients for crop rotation. Furthermore, Halophilic bacteria such as *Halovivax* and *Halorussus* thrive in saline-alkali soils or soils impacted by salinization. Through their physiological and metabolic activities, they can adapt to high-salinity environments, thereby helping to mitigate soil salinization to some extent. The presence of these dominant microorganisms within rotation systems can effectively enhance soil properties, ultimately leading to improved yield and quality of crops such as potatoes.

### LEfSe discriminant analysis

#### LEfSe analysis of soil bacteria between different treatments

**FIGURE 5:**
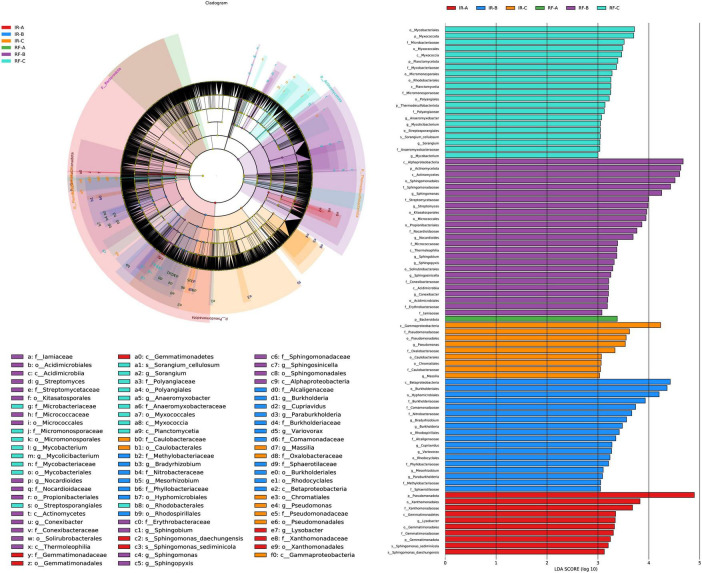
LEfSe analysis of soil bacteria under different treatments. Different colors in **(A)** represent different groups, and nodes of different colors represent the microbial groups that play an important role in the group represented by the color. The yellow nodes represent microbial groups that do not play an important role in different groups. The name of the species represented by the English letters in the diagram shows the species at the family classification level in the legend on the right side. The LDA value distribution histogram in **(B)** shows the species with LDA Score greater than the set value (default setting is 3), that is, the species with statistical differences between groups. Species with significant differences in abundance in different groups were displayed, and the length of the histogram represented the influence of different species (LDA Score).

The LEfSe multi-level species discrimination method is employed to identify unique biomarkers for each group (Figure 5A). Additionally, it effectively highlights the groups exhibiting significant differences in abundance (*p* < 0.05), as well as their influence values (LDA > 3), presented in the LDA value distribution histogram (Figure 5B). In the irrigation system, the number of bacterial biomarkers identified in the IR-A, IR-B, and IR-C treatments was 10, 18, and 9, respectively, with IR-B exhibiting the highest number of markers. The most influential differential bacteria in IR-A were primarily classified within Pseudomonadota (phylum) (*p* < 0.05). The key differential bacteria in IR-B encompassed Betaproteobacteria (class), along with Burkholderiales (order) and Hyphomicrobiales (order) (*p* < 0.05). The most significant differential bacteria in IR-C were classified under Gammaproteobacteria (class) (*p* < 0.05). In the rain-fed system, a greater number of bacterial biomarkers were observed in the RF-B and RF-C treatments, with counts of 25 and 21, respectively. The most abundant markers in RF-B comprised Alphaproteobacteria (class), Actinomycetota (phylum), Actinomycetes (class), Sphingomonadales (order), Sphingomonadaceae (family), and *Sphingomonas* (genus) (*p* < 0.05). In RF-C, the influential bacterial taxa included Mycobacteriales (order) and Myxococcota (phylum) (*p* < 0.05). In contrast, RF-A contained only one biomarker, which was classified as Bacteroidota (phylum) (*p* < 0.05). Significant differences in bacterial biomarkers were observed between continuous cropping and rotation practices, suggesting that the bacterial community in the RF system exhibited greater diversity compared to that in the IR system.

In the IR system, Betaproteobacteria are involved in the oxidation processes of nitrate-nitrite-nitrate, playing a significant role in maintaining the nitrogen balance in soil and plant systems. Additionally, Betaproteobacteria can decompose organic carbon compounds in the soil into smaller molecular carbon compounds, participating in the turnover of carbon in the soil and affecting the dynamic balance of soil organic carbon pools. Hyphomicrobiales and Gammaproteobacteria can play important roles in soil phosphorus and sulfur cycling. In the RF system, Actinomycetota are capable of decomposing organic matter in the soil, releasing essential nutrients such as nitrogen, phosphorus, and potassium, and converting them into forms that are absorbable by plants, thereby enhancing soil fertility. Therefore, in the rotation system, these differential bacterial groups may play crucial roles in maintaining soil ecological balance and promoting crop growth.

#### LEfSe analysis of soil fungi between different treatments

**FIGURE 6:**
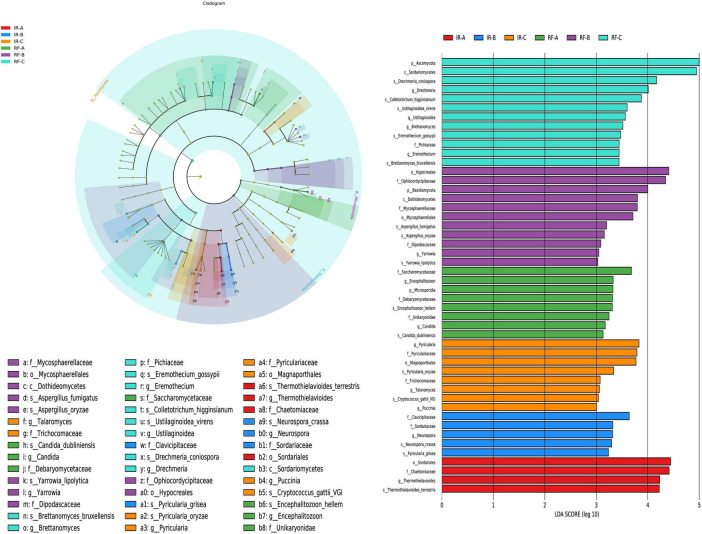
The LefSe analysis of fungi at different degeneration level. Different colors in **(A)** represent different groups, and nodes of different colors represent the microbial groups that play an important role in the group represented by the color. The yellow nodes represent microbial groups that do not play an important role in different groups. The name of the species represented by the English letters in the diagram shows the species at the family classification level in the legend on the right side. The LDA value distribution histogram in **(B)** shows the species with LDA Score greater than the set value (default setting is 3), that is, the species with statistical differences between groups. Species with significant differences in abundance in different groups were displayed, and the length of the histogram represented the influence of different species (LDA Score).

The LEfSe multi-level species discrimination method is employed to identify unique biomarkers for each group (Figure 6A). Additionally, it effectively highlights the groups exhibiting significant differences in abundance (*p* < 0.05), as well as their influence values (LDA > 3), presented in the LDA value distribution histogram (Figure 6B). In the irrigation system, the number of fungal differential groups identified was 4 in IR-A, 5 in IR-B, and 8 in IR-C treatments, respectively. In IR-A, the differential fungi that exhibited significant influence included Sordariales (order) and Chaetomiaceae (family) (*p* < 0.05). In IR-B, Clavicipitaceae (family) emerged as the predominant differential fung (*p* < 0.05). Meanwhile, in IR-C, the influential differential fungi comprised *Pyricularia* (genus), Pyriculariaceae (family) and Magnaporthales (order) (*p* < 0.05). In the rain-fed system, the number of differential fungal groups identified was 8 in RF-A, 11 in RF-B, and 12 in RF-C, respectively. In RF-A, Saccharomycetaceae (family) was recognized as the differential fungi with a significant impact (*p* < 0.05). In RF-B, Hypocreales (order) and Ophiocordycipitaceae (family) were found to be the most influential differential fungi (*p* < 0.05). Furthermore, in RF-C, Ascomycota (phylum) and Sordariomycetes (class) represented the key differential fungi with considerable impact (*p* < 0.05). There were significant differences in bacterial biomarkers between continuous cropping and rotation. Compared with the irrigation system, the rain-fed system contained more abundant fungal differential groups.

In the IR system, Clavicipitaceae can form symbiotic relationships with plants, enhancing their resistance to environmental stresses. Some fungal species within the order Hypocreales are common biocontrol agents that can inhibit the growth of plant pathogens, thereby reducing the reliance on chemical pesticides. In the RF system, Ascomycota and Sordariomycetes play a crucial role in decomposing organic matter in the soil, which is essential for improving soil fertility and promoting the material cycling and energy transformation within the ecosystem.

#### LEfSe analysis of soil archaea between different treatments

**FIGURE 7:**
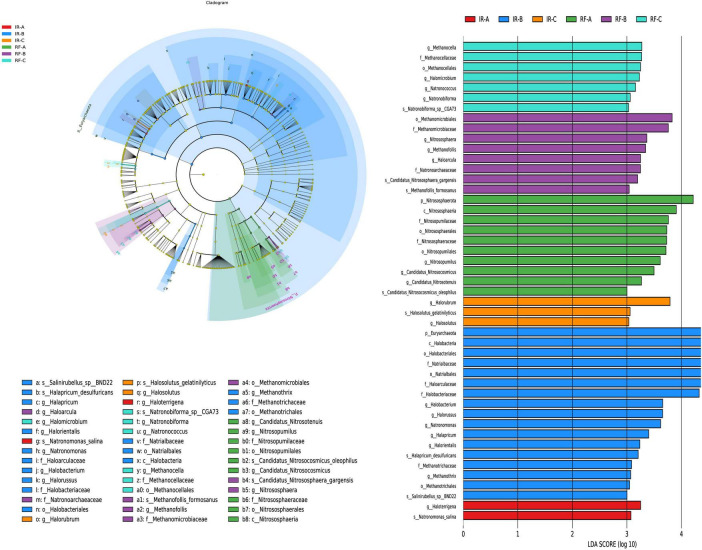
The LefSe analysis of archaea at different degeneration level. Different colors in **(A)** represent different groups, and nodes of different colors represent the microbial groups that play an important role in the group represented by the color. The yellow nodes represent microbial groups that do not play an important role in different groups. The name of the species represented by the English letters in the diagram shows the species at the family classification level in the legend on the right side. The LDA value distribution histogram in **(B)** shows the species with LDA Score greater than the set value (default setting is 3), that is, the species with statistical differences between groups. Species with significant differences in abundance in different groups were displayed, and the length of the histogram represented the influence of different species (LDA Score).

The LEfSe multi-level species discrimination method is employed to identify unique biomarkers for each group (Figure 7A). Additionally, it effectively highlights the groups exhibiting significant differences in abundance (*p* < 0.05), as well as their influence values (LDA > 3), presented in the LDA value distribution histogram (Figure 7B).

In the irrigation system, the IR-B treatment exhibited the highest diversity of archaeal differential groups, totaling 17. The influential archaea identified in IR-B included Euryarchaeota (phylum), Halobacteria (class), and Halobacteriales (order) (*p* < 0.05). In contrast, the IR-A treatment displayed only 2 differential archaea, which were Haloarrigena (genus) and *Natronomonas salina* (species) (*p* < 0.05). Additionally, there were only 3 difference archaea in IR-C treatment, and the difference archaea with great influence in IR-C was Halorubrum (genus) (*p* < 0.05). In the rain-fed system, the number of differential archaeal groups identified was 10 in RF-A, 8 in RF-B, and 7 in RF-C, respectively. In RF-A, Nitrososphaerota (phylum) was noted as the predominant influential archaeon (*p* < 0.05). In RF-B, Methanomicrobiales (order) and Methanomicrobiaceae (family) emerged as the key influential archaea (*p* < 0.05). In RF-C, several influential archaea were identified, including Methanocella (genus), Methanocellaceae (family), Methanocellales (order), and Halomicrobium (genus) (*p* < 0.05).

In the IR system, Euryarchaeota play an important role in the carbon cycle and in adapting to extreme environments such as aridity and high temperatures. Halobacteria, as representatives of extreme halophilic archaea, are significant in maintaining the balance and cycling of sulfur elements in high-salinity ecosystems. In the RF system, the differential microbial groups are primarily dominated by methanogenic archaea, which can convert CO_2_ and H_2_ produced from the decomposition of organic matter in the soil into methane, thus participating in the soil carbon cycle and making important contributions to maintaining soil ecological balance.

Significant differences were observed in bacterial biomarkers between continuous cropping and crop rotation practices (*p* < 0.05). The number of differential archaeal groups in the various treatments was lower than that of bacterial groups; however, the number of differential fungal groups was found to be only 1. The rain-fed system exhibited a greater abundance of differential fungal groups compared to the irrigation system. This indicates a notable distinction in microbial diversity and composition between the two agricultural systems.

#### KEGG functional annotation and enrichment analysis

**FIGURE 8:**
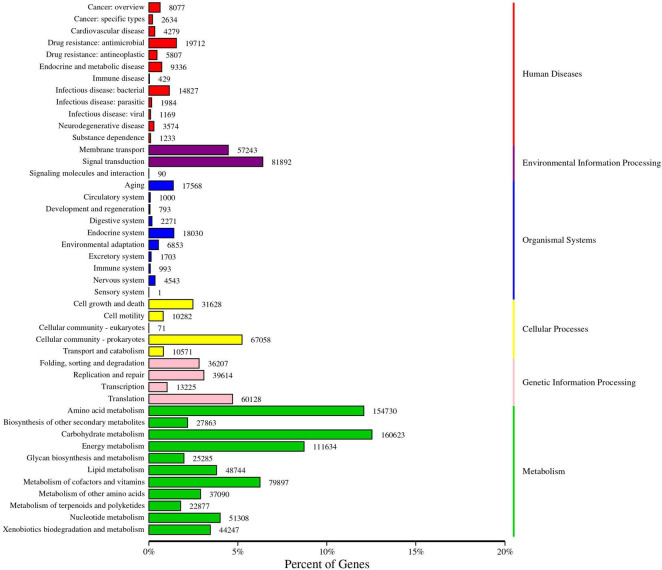
KEGG annotation gene number statistics. The left ordinate is the name of the KEGG secondary classification, and the right ordinate is the name of the KEGG primary classification. The abscissa is the number of genes annotated to this classification and the proportion of the number of genes to the total number of genes annotated.

Soil microbial functional genes were annotated using the KEGG database, as illustrated in Figure 8. The abundance of genes corresponding to the primary KEGG pathways was categorized into six subsystems. The proportions of annotated genes in these categories were as follows: Metabolism (60.01%), Environmental Information Processing (10.72%), Genetic Information Processing (10.15%), Cellular Processes (9.41%), Human Diseases (5.54%), and Organismal Systems (4.16%). Furthermore, the annotation results for the KEGG secondary functional genes revealed that the proportion of annotated genes ranged from 0.95 to 12.56%. These secondary functional genes primarily encompassed carbohydrate metabolism, amino acid metabolism, energy metabolism, signal transduction, and translation, among other processes.

**FIGURE 9:**
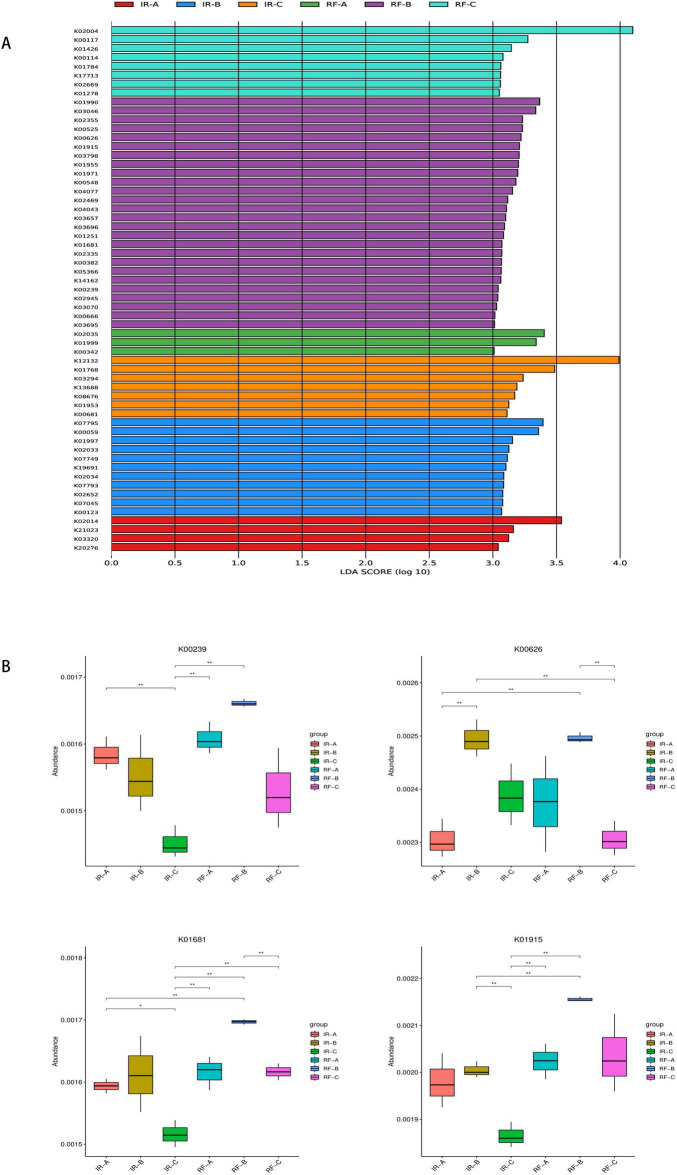
Analysis of KEGG functional differences between groups. The LDA value distribution histogram in **(A)** shows the species with a LDA score greater than the set value (default setting is 3), that is, biomarkers with statistical differences between groups. The length of the histogram represents the influence of the differential KEGG function (LDA score). **(B)** A KEGG function box line diagram with significant differences. The horizontal line in **(B)** represents two groups with significant differences, and no means that there is no difference between the two groups. “*” means significant difference between the two groups (*q* < 0.05), “**” means extremely significant difference between the two groups (*q* < 0.01), “****” means extremely significant difference between the two groups (*q* < 0.001).

**FIGURE 10:**
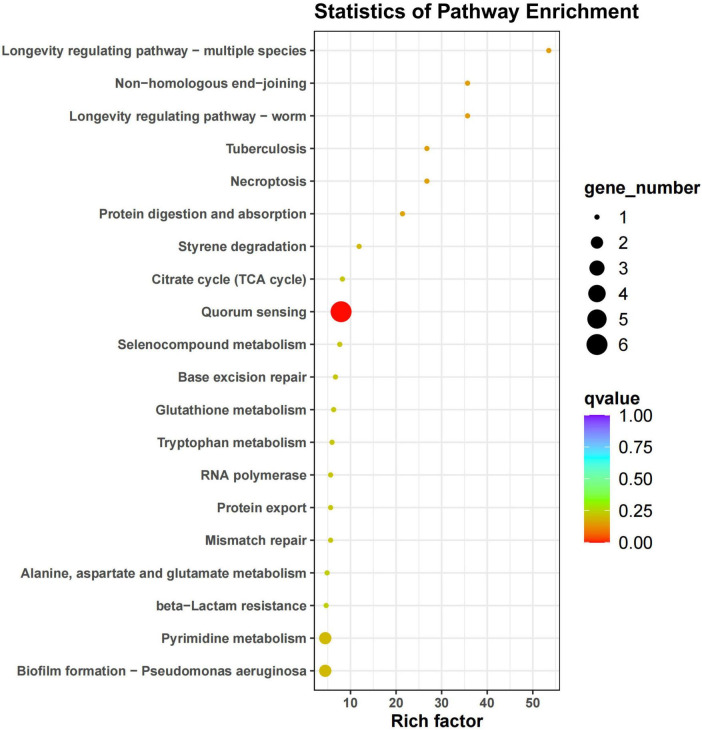
KEGG enrichment map of differential genes. The horizontal axis represents the enrichment factor, while the vertical axis represents the pathway. Different colors indicate different adjusted *q*-values, with blue representing higher values and red representing lower values, indicating increasing significance of enrichment. The size of the origin represents the number of genes enriched in this pathway.

LEfSe analysis of KEGG functions across different treatment groups revealed distinct functional gene profiles, as presented in Figure 9A. The genes associated with carbohydrate metabolism included K00117, K00114, K01784, K00626, K001915, K01681, K00382, K00239, and K00123. In contrast, the genes related to amino acid metabolism comprised K01426, K00626, K01915, K01955, K00548, K01251, K00382, and K01953. These genes were identified as differential genes within the IR-B, IR-C, RF-B, and RF-C treatments. According to the KEGG pathway database, K00239, K00626, K01681, and K01915 were found to participate in three crucial metabolic pathways related to carbon and nitrogen: Carbohydrate Metabolism (map00020), Carbon Fixation Pathways in Prokaryotes (map00720), and Nitrogen Metabolism (map00910). In the Carbohydrate Metabolism pathway (map00020), genes K00239 and K01681 are involved in the citrate cycle. Within the Carbon Fixation Pathways in Prokaryotes pathway (map00720), K00239 is involved in the 3-hydroxypropionate bicycle in carbon fixation. Additionally, K00626 is involved in the hydroxypropionate-hydroxybutylate cycle. In the Nitrogen Metabolism pathway (map00910), gene K01915 is involved in glutamate metabolism in nitrogen metabolism pathway. Under rotation systems, the abundance of K00239, K00626, K01681, and K01915 was significantly higher compared to that observed in continuous cropping systems (*p* < 0.05) (Figure 9B and Supplementary Figure 1). This finding suggests that crop rotation may enhance the functional capacity of soil microbial communities in terms of carbon and nitrogen metabolism. The results of the KEGG enrichment analysis (Figure 10) indicated that 20 significantly enriched pathways were identified. Among these pathways, one gene associated with the tricarboxylic acid cycle reaction in the carbon metabolism pathway was K01681. The enrichment results for gene K01681 were consistent with the previously mentioned KEGG annotation results. In the carbon metabolism pathways, the expression and interactions of key differential genes are crucial for enhancing crop yield and quality. This provides strong evidence for further exploring the mechanisms by which crop rotation alleviates continuous cropping obstacles.

#### RDA analysis of the relationships between soil microorganisms and yield, quality

**FIGURE 11:**
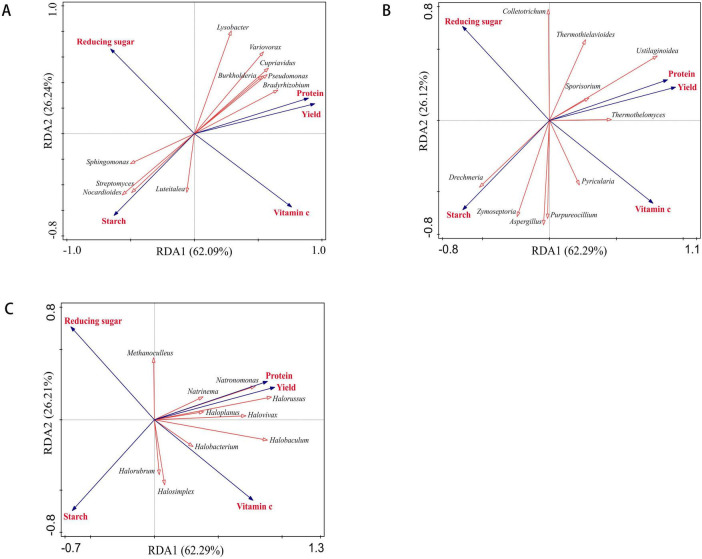
RDA analysis of soil microorganisms and yield, quality. **(A)** RDA analysis of bacteria and yield, quality; **(B)** RDA analysis of fungi and yield, quality; **(C)** RDA analysis of archaea and yield, quality; red arrows represent microbial communities, and blue arrows represent yield and quality. *p* < 0.05 means significant correlations between two properties.

At the genus level, the Redundancy Analysis (RDA) results indicated a strong relationship between soil microorganisms and potato yield and quality. The analysis revealed that the cumulative interpretation rate for bacteria was 88.33%, for fungi it was 88.41%, and for archaea it was 88.50% (Figure 11). Among the 10 dominant bacterial communities identified, *Bradyrhizobium* and *Burkholderia* exhibited a significant positive correlation with potato yield (*p* < 0.05), while Streptomyces demonstrated a significant positive correlation with starch content (*p* < 0.01) (Supplementary Table 3 and Figure 11A). Furthermore, LEfSe analysis revealed that *Bradyrhizobium* and *Burkholderia* were the differential bacterial taxa in the IR-B treatment, whereas *Streptomyces* was identified as the differential bacterium in the RF-B treatment. The relative abundance of these bacterial communities was significantly higher in these treatments compared to those under continuous cropping systems (*p* < 0.05). Among the 10 dominant fungal communities, *Ustilaginoidea* was significantly positively correlated with yield and protein (*p* < 0.01), and significantly negatively correlated with starch (*p* < 0.01) (Supplementary Table 3 and Figure 11B). It was a differential fungal in RF-C treatment, and its abundance was significantly higher than that of continuous cropping (*p* < 0.05). Among the 10 dominant archaeal communities examined, *Halorussus* demonstrated a significant positive correlation with yield and vitamin C content (*p* < 0.01), as well as a significant positive correlation with protein levels (*p* < 0.05). Conversely, it exhibited a significant negative correlation with reducing sugar content (*p* < 0.01). Additionally, *Halobacterium* was found to be significantly positively correlated with both yield and protein (*p* < 0.01), and it also showed a significant positive correlation with vitamin C (*p* < 0.05) (Figure 11C and Supplementary Table 3).

The results indicate that the aforementioned differential microbial communities are key factors influencing the enhancement of potato yield and quality. These microbial populations significantly contribute to improving rhizosphere soil fertility, thereby fostering a more conducive environment for plant growth. Furthermore, the analysis of community structure and the mechanisms underlying soil fertility in the context of crop rotation restoration offers valuable insights.

#### Association network analysis of soil microbial phylum level

**FIGURE 12:**
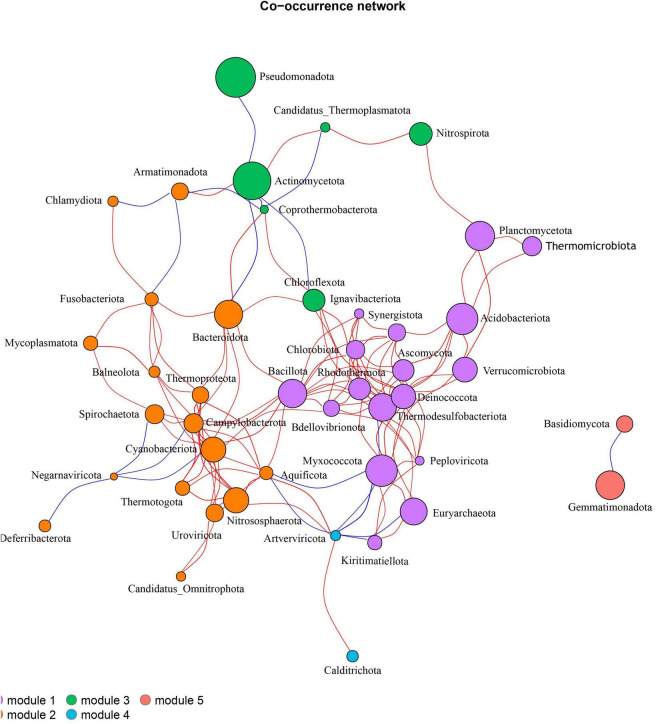
Association network analysis of soil microbial community at phylum level. Each node represents a species. The larger the node, the higher the relative abundance of the species. Different colors represent different modules. The red line indicates positive correlation, and the blue line indicates negative correlation.

Based on the correlation relationships between species, a top 50 microbial community association network analysis was conducted at the phylum level (Figure 12). The results indicate that there are interactions among soil microbes at the phylum level across six treatments. The network has an average degree of 5.82, a clustering coefficient of 0.47, a network diameter of 6, a modularity of 0.42, and an average path length of 2.68, with a total of 128 edges, suggesting that the network exhibits a certain degree of clustering and connectivity. These microbial communities are divided into five distinct modules. Module 1 contains 17 microbial communities, where Thermodesulfobacteriota is negatively correlated with Myxococcota, while the remaining microbes exhibit positive correlations among themselves. In Module 2, Armatimonadota is negatively correlated with Fusobacteriota and Chrysiogenota, and Negarnaviricota is negatively correlated with Deferribacterota, Spirochaetota, Cyanobacteriota, and Campylobacterota, with all other microbes showing positive correlations. In Module 3, Actinomycetota is negatively correlated with Pseudomonadota, Chloroflexota, and Coprothermobacterota, while it is positively correlated with Nitrospirota and Thermoplasmatota. Modules 4 and 5 each contain two microbial communities, where Artverviricota and Calditrichota are positively correlated, while Gemmatimonadota is negatively correlated with Basidiomycota.

The positive and negative correlations among different microbial communities indicate certain synergistic or antagonistic effects within the soil ecosystem. Myxococcota, Bacillota, Acidobacteriota, Verrucomicrobiota, and Ascomycota are involved in the decomposition of organic matter in the soil, thereby releasing nutrients available to plants. Planctomycetota, Thermodesulfobacteriota, Cyanobacteriota, and Nitrososphaerota play a crucial role in the nitrogen cycle, collaboratively participating in soil nitrogen cycling and increasing nitrogen supply in the soil. Some species within Cyanobacteriota possess nitrogen-fixing capabilities, allowing them to convert atmospheric nitrogen into ammonia, providing a nitrogen source for themselves and the surrounding environment. In contrast, Campylobacterota and Spirochaetota are heterotrophic bacteria that rely on organic or inorganic nitrogen sources from the environment for growth. When nitrogen sources in the soil are limited, the nitrogen-fixing ability of Cyanobacteriota enhances its nitrogen supply, which may restrict the nitrogen source availability for Campylobacterota and Spirochaetota, leading to competitive relationships that manifest as negative correlations. This synergistic and antagonistic relationship among microorganisms plays an important role in maintaining the balance and stability of the soil ecosystem.

#### Binning analysis based on metagenomic data assembly

Following the binning assembly of 18 metagenomic datasets, a total of 107 bins were generated. From this initial set, bins exhibiting an integrity greater than 70% and a pollution degree of less than 10% were selected for further genome assembly. As a result, 11 metagenome-assembled genomes (MAGs) were obtained. The average integrity of these MAGs was found to be 82.68%, while the pollution degree averaged at 5.43%. The overall quality score of the assembled MAGs was determined to be 65.06% (Supplementary Table 4).

**FIGURE 13:**
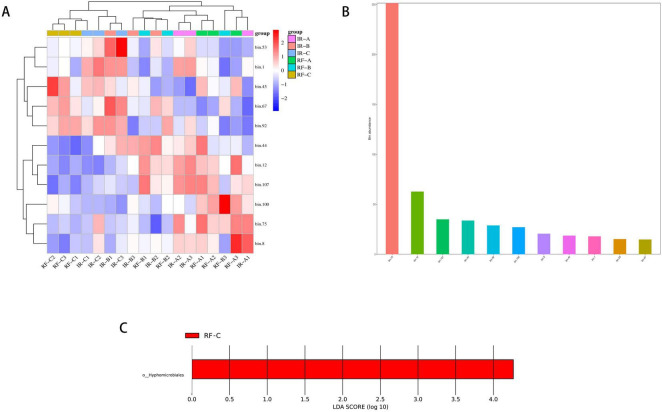
Binning analysis of metagenomic data assembly. **(A)** The bins abundance heat map, which vertically represents the clustering of a certain bins in different samples. The closer the distance is, the shorter the branch length is, indicating that the bins composition and abundance of the samples are more similar. Horizontally represents the clustering of a certain sample in bins. Similar to vertical clustering, the closer the distance is, the shorter the branch length is, indicating that the composition of bins is more similar among samples. **(B)** The bins abundance histogram, the vertical axis represents the abundance of bins, and the horizontal axis is different bins; **(C)** the LDA value distribution histogram for significantly different species, with screening criteria of *p* < 0.05 and LDA score > 3. The vertical axis represents the classification units with significant differences between groups, while the horizontal axis visually displays the logarithmic LDA scores for each classification unit using a bar chart. The color of the bars indicates which classification unit corresponds to the sample group with higher abundance.

Among the 11 MAGs, bins75 exhibited the highest abundance (Figure 13B) and is primarily classified within the Nitrososphaerota phylum, playing a crucial role in the nitrogen cycle. The abundance heat map of the MAGs revealed that the 11 MAGs could be categorized into two distinct groups across different treatments, with the abundance of MAGs in the RF-C treatment being similar to that observed in the IR-C treatment (Figure 13A). Furthermore, the results from the LEfSe analysis indicated that the relative abundance of Hyphomicrobiales in the RF-C treatment was significantly higher compared to the other treatments (Figure 13C) (*p* < 0.05). Hyphomicrobiales are known to form symbiotic relationships with plants, providing essential nitrogen through nitrogen fixation, thereby promoting plant growth and development.

## Discussion

### Effects of rotation on soil microbial abundance

Many studies have demonstrated that the occurrence of continuous cropping obstacles is closely linked to the types and quantities of soil microorganisms (Wang Y. et al., 2023; Hu et al., 2022; Zhang J. X. et al., 2022). Liu et al. (2020) demonstrated that long-term continuous soybean cropping led to an increased relative abundance of soil fungi such as *Mortierella*, *Guehomyces*, and *Alternaria* when compared to soybean rotation practices. Xu’s research indicated that the relative abundance of beneficial bacteria, specifically *Bacillus* and *Nocardioides*, decreased following continuous cropping of potatoes. Furthermore, the overall abundance of the bacterial community declined as the duration of cropping increased. Notably, the relative abundance of *Bacillus* reached its lowest point after 6 years of continuous cropping. In comparison to the abundance observed after 1 year of continuous cropping, the relative abundance of *Nocardioides* decreased by 2.34% after 7 years (Xu et al., 2024).

In order to alleviate the issues caused by continuous cropping, an increasing number of people are using large amounts of chemical fertilizers and pesticides to boost potato yields. However, this practice may lead to the destruction of a healthy soil ecosystem (Larkin et al., 2021). Reasonable rotation is one of the effective ways to alleviate soil continuous cropping obstacles. For example, rice-potato rotation significantly increased the abundance of 13 types of soil functional microbial communities, while continuous cropping only raised the abundance of 4 types, including chitin-decomposing bacteria and plant pathogenic fungi (Zhou et al., 2023). Research by Qin et al. (2022) indicated that in the oat-bean-potato-oat and bean-potato-oat-bean treatments, the relative abundances of *Devosia*, *Aeromicrobium*, *Paraphoma*, and *Papiliotrema* were significantly higher than those in the potato continuous cropping treatment, whereas the relative abundances of *Pseudomonas*, *Colletotrichum*, *Plectosphaerella*, *Fusarium* and *Verticillium* increased in the continuous cropping treatment. Sanzovon’s study demonstrated that implementing crop rotation practices can significantly enhance the population of *Bradyrhizobium* in the soil (Sanzovo et al., 2023). Xiang’s study demonstrated that ryegrass rotation significantly altered soil biochemical properties and bacterial communities, particularly increasing the abundance of beneficial bacteria such as *Pseudomonas* (Xiang et al., 2024). This is different from Qin’s research findings.

In this study, a comprehensive analysis of microbial diversity was conducted. The results indicated that the implementation of a rotation system significantly enhanced the relative abundance of specific dominant bacteria, fungi, and archaea within the soil (*p* < 0.05). Furthermore, this rotational approach had a marked positive effect on the overall soil ecosystem, contributing to improvements in both crop yield and quality. In comparison to the continuous cropping mode, the integrated rotation-breeding (IR-B) system significantly increased the relative abundance of both *Bradyrhizobium* and *Halorussus* (*p* < 0.05). The plant hormones synthesized by *Bradyrhizobium* are known to play a crucial role in regulating plant growth and development, thereby enhancing crop performance. Additionally, *Halorussus* contributes to the decomposition and transformation of organic matter, as well as the metabolism of nitrogen, which is essential for maintaining soil fertility and promoting nutrient availability for plants. RDA demonstrated that both *Bradyrhizobium* and *Halorussus* exhibited a positive correlation with crop yield. Furthermore, *Halorussus* was found to be positively correlated with vitamin C content, while displaying a negative correlation with reducing sugar content. LEfSe indicated that *Bradyrhizobium* and *Halorussus* were differential microbial communities in the IR-B treatment. The IR-B treatment significantly improved both the yield and vitamin C content of potatoes while reducing the levels of reducing sugars (*p* < 0.05). This suggests that the increased abundance of *Bradyrhizobium* and *Halorussus* in the soil positively influences crop yield and quality. These findings provide a theoretical foundation for further investigation into the mechanisms by which crop rotation mitigates the challenges associated with continuous cropping. Rotation reduced the relative abundance of *Bradyrhizobium*, *Pseudomonas* and *Cupriavidus* under RF planting mode, which was in contrast to the results under IR planting mode (*p* < 0.05). The results of IR model were consistent with Xiang’s study. We believe that the reason for this result may be that under the RF planting mode, the soil moisture content is relatively low, resulting in a decrease in cell enzyme activity, a blocked metabolic process, and a decrease in the number of organic substances in the root exudates as microbial carbon and nitrogen sources, thereby inhibiting the growth and reproduction of these bacteria. Therefore, we need to further explore how soil environmental factors regulate the change of microbial community structure under rain-fed mode, so as to more fully reveal the internal mechanism of rotation to reduce continuous cropping obstacles.

### Effect of rotation on soil microbial diversity index

The number and diversity of microorganisms in soil are crucial factors for enhancing soil fertility, promoting nutrient decomposition and transformation, increasing crop yields, and improving crop quality (Yin et al., 2020). Microorganisms play an indispensable role in soil nutrient cycling, and microbial diversity indices serve as indicators of soil ecological health and fertility. Zhang L. et al. (2020) reported that both the Shannon and Simpson indices for bacteria significantly increased following rice-rapeseed rotation. Similarly, Hu et al. (2022) found that rotating potatoes with peas and wheat led to a notable increase in fungal diversity indices. Liu et al. (2015) also indicated a significant increase in the Shannon index for fungi after potato rotation, with a marked difference in fungal community composition compared to continuous cropping. Additionally, Liu H. et al. (2019) demonstrated that the Shannon and Chao1 indices in the rhizosphere soil of cassava rotations were higher than those observed in continuous cropping scenarios. Conversely, Deng (2018) reported that the number of fungi following the rotation of cherry tomatoes was significantly lower than that in continuous cropping; however, the fungal diversity did not show a significant difference.

The results of α diversity in this study indicated that potato-maize rotation significantly increased the Shannon index of soil bacteria under the irrigated planting pattern (*p* < 0.05), suggesting improved microbial richness and evenness, which aligns with Zhang’ s findings. However, the Simpson index for the IR-C treatment was notably lower than that for the IR-A treatment after rotation (*p* < 0.05), contradicting Zhang’s conclusions. This discrepancy may stem from the fact that the Simpson index reflects community diversity, with higher values indicating lower diversity. It primarily accounts for the dominance of the most prevalent species in the community, making it less sensitive to rare species. In continuous cropping systems, the limited variety of root exudates from a single crop results in fewer microbial species being able to utilize these exudates for growth, leading to the formation of dominant species. This, in turn, reduces community diversity and increases the Simpson index. Conversely, crop rotation introduces a variety of root exudates from different crops, creating favorable conditions for a wider range of microorganisms. This increases microbial abundance and diminishes the relative dominance of any single species, thereby lowering the Simpson index. Under the rain-fed planting mode, the RF-B treatment significantly increased the Simpson index of soil bacteria (*p* < 0.05), which is consistent with Zhang’s conclusions; however, it decreased the Shannon index, which contradicts Zhang’s findings. This may be attributed to the drought conditions leading to reduced soil moisture, allowing drought-tolerant microbial groups to dominate the community while inhibiting other microorganisms. As a result, the uniformity of microbial species decreases, contributing to a lower Shannon index. In this study, no significant differences were observed in the Shannon and Simpson indices of the fungal community between continuous cropping and rotation (*p* > 0.05), which contrasts with the findings of Hu and Liu but aligns with Deng’s results. The changes in fungal community diversity are closely related to the soil’s ecological environment and microbial competition. Since there was no significant difference in soil pH between continuous cropping and rotation, the maize rotation had minimal impact on soil pH. Consequently, the fungal community structure likely remained unaffected by pH changes, resulting in stable Shannon and Simpson indices.

The results of PCoA indicated significant differences in the soil bacterial, fungal, and archaeal communities between continuous cropping and rotation systems (*p* < 0.05). Wang Y. et al. (2023) conducted NMDS to examine the β diversity of soil fungal communities under various potato rotation patterns, revealing notable differences in fungal community structures between rotation and continuous cropping. Additionally, Wang et al. found clear community separation in both bacterial and fungal structures between mushroom-tobacco rotation and tobacco continuous cropping soils, further supporting the findings of this study (Wang X. et al., 2023).

### KEGG function analysis of microbial community

Microorganisms are vital components of soil ecosystems, playing an irreplaceable role in essential functions such as nutrient cycling and metabolism (Wang and Wang, 2020). Crop rotation promotes the synthesis of metabolites and supports adequate energy metabolism, which is crucial for enhancing plant tolerance (Zhang K. L. et al., 2022). Tang et al. (2020) reported the presence of numerous metabolism-related genes within the soil microbial community in dolomite coastal areas, noting that the metabolic pathways associated with these genes are closely linked to various soil environmental factors. Furthermore, Pang et al. (2021) observed that while the functional metabolic pathways of microorganisms under different treatments were generally similar, the gene sequences within specific pathways differed, suggesting that distinct microbial species may engage in unique metabolic processes under varying environmental conditions. This diversity contributes to the stability of ecosystem functions.

In this study, the LEfSe analysis of KEGG functions revealed differences in gene presence among various treatments. These genes were involved in specific reactions within carbon and nitrogen metabolic pathways. For instance, genes K00239 and K01681 were associated with the tricarboxylic acid cycle, while K01915 was linked to the nitrogen fixation process. The activation of these metabolic pathways is closely related to crucial ecological functions such as carbon and nitrogen cycling, which are significant for maintaining soil fertility and promoting plant growth. Zhang et al. found that several genes associated with microbial carbon fixation, such as ACAT, IDH1, GAPDH, rpiA, and rbcS, were significantly enriched in a wheat-soybean rotation system. In our study, we identified gene K00626 (Acat) as being involved in three key metabolic pathways: Carbohydrate metabolism (map00020), Carbon fixation pathways in prokaryotes (map00720), and Nitrogen metabolism (map00910). K00626 emerged as a differential gene within the rotation system, corroborating the findings of Zhang et al.

In addition, we performed KEGG functional annotation of rhizosphere soil microorganisms after potato continuous cropping and potato-maize rotation. The results show that the 45 annotated pathways were divided into six primary metabolic pathways: Metabolism, Environmental information processing, Genetic information processing, Cellular processes, Human Diseases and Orginismal System. The number of functional genes of Carbohydrate metabolism, Amino acid metabolism and Energy metabolism is relatively high, indicating that they play a key role in plant growth, development and metabolism. Through the transport and metabolism of amino acids, phosphorus and inorganic ions, there is a certain change in the permeability of the outer membrane of the cell, thereby reducing the impact of the external environment on the cell (Feng et al., 2018; Yang et al., 2019), which shows that soil microorganisms have a certain ability to adapt to adversity stress and environmental interference. In this study, potato-maize rotation increased soil total nitrogen content, probably because Carbohydrate metabolism and Amino acid metabolism regulated the expression of more genes and helped to improve plant resistance (Souza et al., 2015; Zhang K. L. et al., 2022; Zhang Y. P. et al., 2022). In the KEGG functional annotation, a total of four differential genes (K00239, K00626, K01681, and K01915) were found to participate in three key metabolic pathways related to carbon and nitrogen. Among them, the gene K01681 was enriched in the tricarboxylic acid cycle reaction of the carbon metabolism pathway. Our research found that potato-maize rotation significantly increased the abundance of gene K01681 (*p* < 0.05). The elevated expression level of this gene may directly impact carbon source utilization efficiency, thereby playing a crucial regulatory role in enhancing crop yield and quality. Therefore, in-depth studies on the K01681 gene and the functions of its encoded protein are of great significance for revealing the regulatory mechanisms of carbon metabolism in alleviating crop continuous cropping obstacles.

## Conclusion

Under the irrigated (IR) mode, compared to IR-A, IR-B significantly increased yield and vitamin C content (*p* < 0.05) while reducing reducing sugar content (*p* < 0.05). In the rain-fed (RF) mode, compared to RF-A, RF-B significantly increased yield and starch content (*p* < 0.05) and also increased vitamin C content (*p* < 0.05). In the IR system, the relative abundance of *Bradyrhizobium* and *Pseudomonas* in the rotation treatment was significantly higher than that in the continuous cropping treatment (*p* < 0.05). In the RF system, *Sphingomonas* and *Streptomyces* showed a significantly higher relative abundance in the RF-B treatment compared to RF-A. Additionally, *Luteitalea* had the highest relative abundance in the RF-C treatment (*p* < 0.05). LEfSe analysis of KEGG functions among the different treatment groups revealed distinct functional genes associated with each treatment. Specifically, genes K00239, K00626, K01681, and K01915 were linked to three critical carbon and nitrogen metabolic pathways: Carbohydrate metabolism, Carbon fixation pathways in prokaryotes, and Nitrogen metabolism. The abundance of genes K00239, K00626, K01681, and K01915 was significantly higher under the rotation systems compared to continuous cropping (*p* < 0.05). This increase may be attributed to crop rotation’ s role in enhancing the relative abundance of beneficial soil microorganisms, promoting the decomposition and transformation of organic matter, and improving the efficiency of carbon and nitrogen metabolism within crops. Consequently, these factors contribute to increased yields and improved quality of potatoes.

## Data Availability

The data presented in the study are deposited in the NCBI repository, accession number PRJNA1243585.
