# Supplementary material for: Effects of rotation corn on potato yield, quality, and soil microbial communities
Source: Front Microbiol. 2025 Apr 16;16:1493333. doi: 10.3389/fmicb.2025.1493333 (PMC12040919; doi:10.3389/fmicb.2025.1493333)
Supplement: Supplementary file 1 [file Table_1.docx]

| Microorganism | Treatment | shannon | | simpson | |
| --- | --- | --- | --- | --- | --- |
|  |  | q value | significance | q value | significance |
| Bacteria | IR-A_IR-B | 0.099 | ns | 0.989 | ns |
|  | IR-A_IR-C | 1.000 | ns | 0.024 | * |
|  | IR-A_RF-A | 0.252 | ns | 0.999 | ns |
|  | IR-A_RF-B | 0.011 | * | 0.054 | ns |
|  | IR-A_RF-C | 0.431 | ns | 0.132 | ns |
|  | IR-B_IR-C | 0.152 | ns | 0.065 | ns |
|  | IR-B_RF-A | 0.988 | ns | 1.000 | ns |
|  | IR-B_RF-B | 0.000 | *** | 0.020 | * |
|  | IR-B_RF-C | 0.904 | ns | 0.318 | ns |
|  | IR-C_RF-A | 0.365 | ns | 0.043 | * |
|  | IR-C_RF-B | 0.007 | ** | 0.000 | *** |
|  | IR-C_RF-C | 0.581 | ns | 0.896 | ns |
|  | RF-A_RF-B | 0.000 | *** | 0.030 | * |
|  | RF-A_RF-C | 0.998 | ns | 0.225 | ns |
|  | RF-B_RF-C | 0.001 | *** | 0.001 | *** |
| Fungi | IR-A_IR-B | 0.894 | ns | 1.000 | ns |
|  | IR-A_IR-C | 0.385 | ns | 0.146 | ns |
|  | IR-A_RF-A | 0.772 | ns | 0.990 | ns |
|  | IR-A_RF-B | 0.302 | ns | 0.180 | ns |
|  | IR-A_RF-C | 1.000 | ns | 0.748 | ns |
|  | IR-B_IR-C | 0.081 | ns | 0.143 | ns |
|  | IR-B_RF-A | 0.244 | ns | 0.989 | ns |
|  | IR-B_RF-B | 0.060 | ns | 0.176 | ns |
|  | IR-B_RF-C | 0.929 | ns | 0.739 | ns |
|  | IR-C_RF-A | 0.977 | ns | 0.341 | ns |
|  | IR-C_RF-B | 1.000 | ns | 1.000 | ns |
|  | IR-C_RF-C | 0.336 | ns | 0.760 | ns |
|  | RF-A_RF-B | 0.941 | ns | 0.406 | ns |
|  | RF-A_RF-C | 0.715 | ns | 0.966 | ns |
|  | RF-B_RF-C | 0.260 | ns | 0.828 | ns |
| Archaea | IR-A_IR-B | 0.461 | ns | 0.925 | ns |
|  | IR-A_IR-C | 0.999 | ns | 1.000 | ns |
|  | IR-A_RF-A | 0.335 | ns | 0.619 | ns |
|  | IR-A_RF-B | 0.992 | ns | 0.913 | ns |
|  | IR-A_RF-C | 0.514 | ns | 0.509 | ns |
|  | IR-B_IR-C | 0.663 | ns | 0.924 | ns |
|  | IR-B_RF-A | 0.017 | * | 0.187 | ns |
|  | IR-B_RF-B | 0.775 | ns | 1.000 | ns |
|  | IR-B_RF-C | 1.000 | ns | 0.955 | ns |
|  | IR-C_RF-A | 0.202 | ns | 0.622 | ns |
|  | IR-C_RF-B | 1.000 | ns | 0.911 | ns |
|  | IR-C_RF-C | 0.717 | ns | 0.506 | ns |
|  | RF-A_RF-B | 0.148 | ns | 0.176 | ns |
|  | RF-A_RF-C | 0.020 | * | 0.049 | * |
|  | RF-B_RF-C | 0.823 | ns | 0.963 | ns |

Supplement Table 1 The significant difference of shannon index and simpson index between different treatments was analyzed.
