# Supplementary material for: Effects of rotation corn on potato yield, quality, and soil microbial communities
Source: Front Microbiol. 2025 Apr 16;16:1493333. doi: 10.3389/fmicb.2025.1493333 (PMC12040919; doi:10.3389/fmicb.2025.1493333)
Supplement: Supplementary file 2 [file Table_2.docx]

Supplementary Table 2 Difference analysis of relative abundance of soil microorganisms at genus level under different treatments

| Microbial species | Name | Treatment | | | | | |
| --- | --- | --- | --- | --- | --- | --- | --- |
|  |  | IR-A | IR-B | IR-C | RF-A | RF-B | RF-C |
| Bacteria | Sphingomonas | 7.39±0.10 a | 6.49±0.48 a | 6.42±0.53 a | 6.99±0.06 b | 8.54±0.18 a | 5.51±0.13 c |
|  | Streptomyces | 4.93±0.02 a | 4.81±0.05 b | 4.88±0.01 ab | 4.96±0.09 b | 5.53±0.11 a | 5.25±0.03 ab |
|  | Bradyrhizobium | 2.20±0.02 b | 2.30±0.01 a | 2.25±0.03 ab | 2.21±0.01 a | 2.02±0.01 b | 2.22±0.01 a |
|  | Luteitalea | 2.02±0.01 a | 2.06±0.02 a | 2.03±0.04 a | 2.07±0.03 b | 2.06±0.07 b | 2.42±0.01 a |
|  | Pseudomonas | 2.01±0.02 b | 2.07±0.02 ab | 2.12±0.03 a | 2.02±0.02 a | 1.82±0.03 b | 2.00±0.02 a |
|  | Burkholderia | 1.55±0.01 a | 1.61±0.01 a | 1.60±0.02 a | 1.56±0.03 a | 1.34±0.01 c | 1.51±0.02 b |
|  | Nocardioides | 1.29±0.07 a | 1.23±0.09 a | 1.30±0.06 a | 1.12±0.01 b | 1.99±0.07 a | 1.41±0.04 b |
|  | Lysobacter | 1.22±0.01 a | 1.14±0.02 a | 1.14±0.01 a | 1.18±0.04 b | 0.98±0.02 a | 0.97±0.01 b |
|  | Variovorax | 1.11±0.01 a | 1.11±0.01 a | 1.08±0.01 a | 1.07±0.02 a | 0.95±0.02 b | 1.00±0.03 b |
|  | Cupriavidus | 0.99±0.01 b | 1.04±0.01 a | 1.03±0.02 ab | 0.99±0.01 a | 0.82±0.02 c | 0.93±0.01 b |
| Fungal | Thermothielavioides | 13.80±0.01 a | 13.33±0.11 b | 12.75±0.14 c | 13.47±0.09 a | 12.86±0.05 b | 13.33±0.09 a |
|  | Drechmeria | 8.65±0.03 a | 8.58±0.02 a | 8.64±0.03 a | 8.64±0.09 b | 8.93±0.02 ab | 9.05±0.12 a |
|  | Colletotrichum | 8.14±0.12 a | 7.94±0.15 a | 8.15±0.04 a | 8.16±0.10 a | 7.78±0.03 b | 8.19±0.09 a |
|  | Purpureocillium | 6.95±0.03 b | 7.19±0.03 a | 7.07±0.09 ab | 7.09±0.13 ab | 7.32±0.06 a | 6.94±0.02 b |
|  | Thermothelomyces | 6.23±0.02 a | 6.39±0.03 a | 6.32±0.10 a | 6.22±0.01 a | 6.25±0.12 a | 6.43±0.08 a |
|  | Pyricularia | 4.96±0.02 b | 5.08±0.01 ab | 5.35±0.14 a | 4.95±0.02 a | 5.10±0.05 a | 5.16±0.10 a |
|  | Aspergillus | 3.75±0.03 a | 3.83±0.11 a | 3.71±0.01 a | 3.67±0.08 b | 4.05±0.03 a | 3.64±0.09 b |
|  | Sporisorium | 3.67±0.08 b | 4.05±0.03 a | 3.64±0.09 b | 3.53±0.01 b | 3.70±0.04 a | 3.76±0.03 a |
|  | Ustilaginoidea | 2.82±0.05 a | 2.90±0.01 a | 2.83±0.06 a | 2.71±0.01 b | 2.64±0.02 b | 2.92±0.08 a |
|  | Zymoseptoria | 2.42±0.01 a | 2.55±0.02 a | 2.53±0.10 a | 2.59±0.09 a | 2.70±0.09 a | 2.42±0.04 a |
| Archaea | Halorubrum | 3.91±0.06 a | 3.84±0.01 a | 3.96±0.01 a | 3.77±0.02 b | 3.95±0.01 a | 3.83±0.02 b |
|  | Halobaculum | 3.45±0.04 a | 3.47±0.01 a | 3.48±0.02 a | 3.33±0.03 a | 3.49±0.09 a | 3.51±0.05 a |
|  | Halovivax | 3.42±0.01 b | 3.53±0.02 a | 3.44±0.02 b | 3.35±0.05 a | 3.39±0.06 a | 3.40±0.02 a |
|  | Natrinema | 3.41±0.05 a | 3.43±0.01 a | 3.49±0.03 a | 3.39±0.01 a | 3.35±0.05 a | 3.44±0.03 a |
|  | Halorussus | 3.09±0.02 b | 3.24±0.01 a | 3.11±0.02 b | 3.00±0.01 b | 2.94±0.03 c | 3.07±0.01 a |
|  | Halobacterium | 3.02±0.01 b | 3.13±0.03 a | 3.02±0.03 b | 2.98±0.02 b | 2.99±0.01 b | 3.10±0.01 a |
|  | Natronomonas | 2.94±0.01 a | 2.95±0.02 a | 2.94±0.02 a | 2.83±0.02 a | 2.87±0.03 a | 2.87±0.01 a |
|  | Haloplanus | 2.04±0.04 a | 2.09±0.02 a | 2.06±0.01 a | 2.04±0.01 a | 2.02±0.05 a | 2.14±0.03 a |
|  | Methanoculleus | 1.98±0.02 a | 1.89±0.06 a | 1.85±0.01 a | 1.91±0.02 a | 1.92±0.03 a | 1.95±0.02 a |
|  | Halosimplex | 1.80±0.03 a | 1.85±0.02 a | 1.84±0.04 a | 1.80±0.02 b | 1.87±0.01 a | 1.85±0.01 a |

Note : The lowercase letters represent the significant difference between treatments ( p < 0.05 ), and the data are expressed as mean ± standard error ( n = 3 ).
