# Supplementary material for: Effects of rotation corn on potato yield, quality, and soil microbial communities
Source: Front Microbiol. 2025 Apr 16;16:1493333. doi: 10.3389/fmicb.2025.1493333 (PMC12040919; doi:10.3389/fmicb.2025.1493333)
Supplement: Supplementary file 3 [file Table_3.docx]

Supplementary Table 3 Significance analysis of soil microorganisms and yield, quality

| Microorganism | Name | Explains(%) | Contribution(%) | pseudo-F | P | P(adj) |
| --- | --- | --- | --- | --- | --- | --- |
| Bacteria | Bradyrhizobium | 32.1 | 32.8 | 4.7 | 0.010 | 0.070 |
|  | Burkholderia | 20.0 | 20.4 | 3.7 | 0.030 | 0.100 |
|  | Luteitalea | 23.1 | 23.6 | 7.4 | 0.004 | 0.040 |
|  | Streptomyces | 9.6 | 9.8 | 4.4 | 0.008 | 0.033 |
|  | Variovorax | 4.7 | 4.8 | 2.7 | 0.066 | 0.132 |
|  | Lysobacter | 2.8 | 2.9 | 1.8 | 0.172 | 0.266 |
|  | Pseudomonas | 1.3 | 1.4 | 0.8 | 0.478 | 0.613 |
|  | Cupriavidus | 3.0 | 3.0 | 2.6 | 0.130 | 0.217 |
|  | Sphingomonas | 1.1 | 1.1 | 0.9 | 0.516 | 0.573 |
|  | Nocardioides | 0.3 | 0.3 | 0.1 | 0.852 | 0.852 |
| Fungi | Ustilaginoidea | 45.9 | 46.7 | 8.5 | 0.004 | 0.040 |
|  | Colletotrichum | 17.1 | 17.4 | 4.1 | 0.024 | 0.120 |
|  | Zymoseptoria | 9.5 | 9.7 | 2.8 | 0.080 | 0.267 |
|  | Sporisorium | 5.4 | 5.5 | 1.7 | 0.170 | 0.425 |
|  | Thermothielavioides | 6.8 | 6.9 | 2.7 | 0.118 | 0.295 |
|  | Aspergillus | 5.5 | 5.6 | 2.8 | 0.074 | 0.200 |
|  | Drechmeria | 4.0 | 4.1 | 2.8 | 0.064 | 0.133 |
|  | Thermothelomyces | 1.8 | 1.8 | 1.3 | 0.336 | 0.420 |
|  | Pyricularia | 1.7 | 1.7 | 1.4 | 0.314 | 0.373 |
|  | Purpureocillium | 0.6 | 0.6 | 0.3 | 0.738 | 0.738 |
| Archaea | Halorussus | 53.4 | 53.5 | 11.4 | 0.002 | 0.010 |
|  | Halobacterium | 10.7 | 10.8 | 2.7 | 0.080 | 0.400 |
|  | Methanoculleus | 7.5 | 7.6 | 2.1 | 0.114 | 0.350 |
|  | Haloplanus | 6.8 | 6.8 | 2.2 | 0.108 | 0.248 |
|  | Natronomonas | 6.5 | 6.5 | 2.6 | 0.078 | 0.190 |
|  | Halobaculum | 2.3 | 2.3 | 0.9 | 0.472 | 0.695 |
|  | Halovivax | 4.6 | 4.6 | 2.2 | 0.150 | 0.250 |
|  | Halorubrum | 4.3 | 4.3 | 3.3 | 0.104 | 0.190 |
|  | Halosimplex | 1.7 | 1.7 | 1.6 | 0.316 | 0.395 |
|  | Natrinema | 2.0 | 2.0 | 8.0 | 0.122 | 0.174 |
