# Supplementary material for: Effects of rotation corn on potato yield, quality, and soil microbial communities
Source: Front Microbiol. 2025 Apr 16;16:1493333. doi: 10.3389/fmicb.2025.1493333 (PMC12040919; doi:10.3389/fmicb.2025.1493333)
Supplement: Supplementary file 4 [file Table_4.docx]

Supplementary Table 4 Filtered bins result table

| Bin ID | Species Annotation Classification | Completeness (%) | Contamination（%） | N50  （bp） | N90  （bp） | Total length （bp） | GC （%） |
| --- | --- | --- | --- | --- | --- | --- | --- |
| bin.1 | k_Bacteria | 70.30 | 2.63 | 10100 | 3933 | 7195176 | 71.20 |
| bin.8 | k_Bacteria | 85.04 | 4.95 | 10200 | 3741 | 28980482 | 63.26 |
| bin.12 | k_Bacteria;p_Pseudomonadota;c_Alphaproteobacteria;o_Sphingomonadales;f_Sphingomonadaceae;g_Sphingomonas | 76.44 | 6.93 | 10124 | 3583 | 14230363 | 66.53 |
| bin.44 | k_Bacteria | 85.00 | 1.71 | 10268 | 3764 | 17126638 | 64.42 |
| bin.45 | k_Bacteria;p_Pseudomonadota;c_Alphaproteobacteria;o_Hyphomicrobiales | 91.30 | 4.42 | 10064 | 3710 | 20231302 | 63.79 |
| bin.53 | k_Bacteria | 89.32 | 4.75 | 10200 | 3710 | 23317905 | 64.49 |
| bin.67 | k_Archaea;p_Nitrososphaerota;c_Nitrososphaeria;o_Nitrososphaerales;  f_Nitrososphaeraceae | 76.94 | 8.31 | 10168 | 3715 | 24673084 | 63.89 |
| bin.75 | k_Archaea;p_Nitrososphaerota;c_Nitrososphaeria;o_Nitrososphaerales;  f_Nitrososphaeraceae | 83.98 | 5.83 | 10039 | 3669 | 26121753 | 62.33 |
| bin.92 | k_Bacteria | 71.43 | 9.10 | 9733 | 3523 | 31149966 | 63.66 |
| bin.100 | k_Archaea;p_Nitrososphaerota;c_Nitrososphaeria;o_Nitrososphaerales;  f_Nitrososphaeraceae | 88.51 | 7.85 | 10806 | 4105 | 9091133 | 65.30 |
| bin.107 | k_Bacteria | 91.21 | 3.30 | 10622 | 3878 | 11977317 | 66.76 |
