# Supplementary figures and images for: Effects of rotation corn on potato yield, quality, and soil microbial communities
Source: Front Microbiol. 2025 Apr 16;16:1493333. doi: 10.3389/fmicb.2025.1493333 (PMC12040919; doi:10.3389/fmicb.2025.1493333)

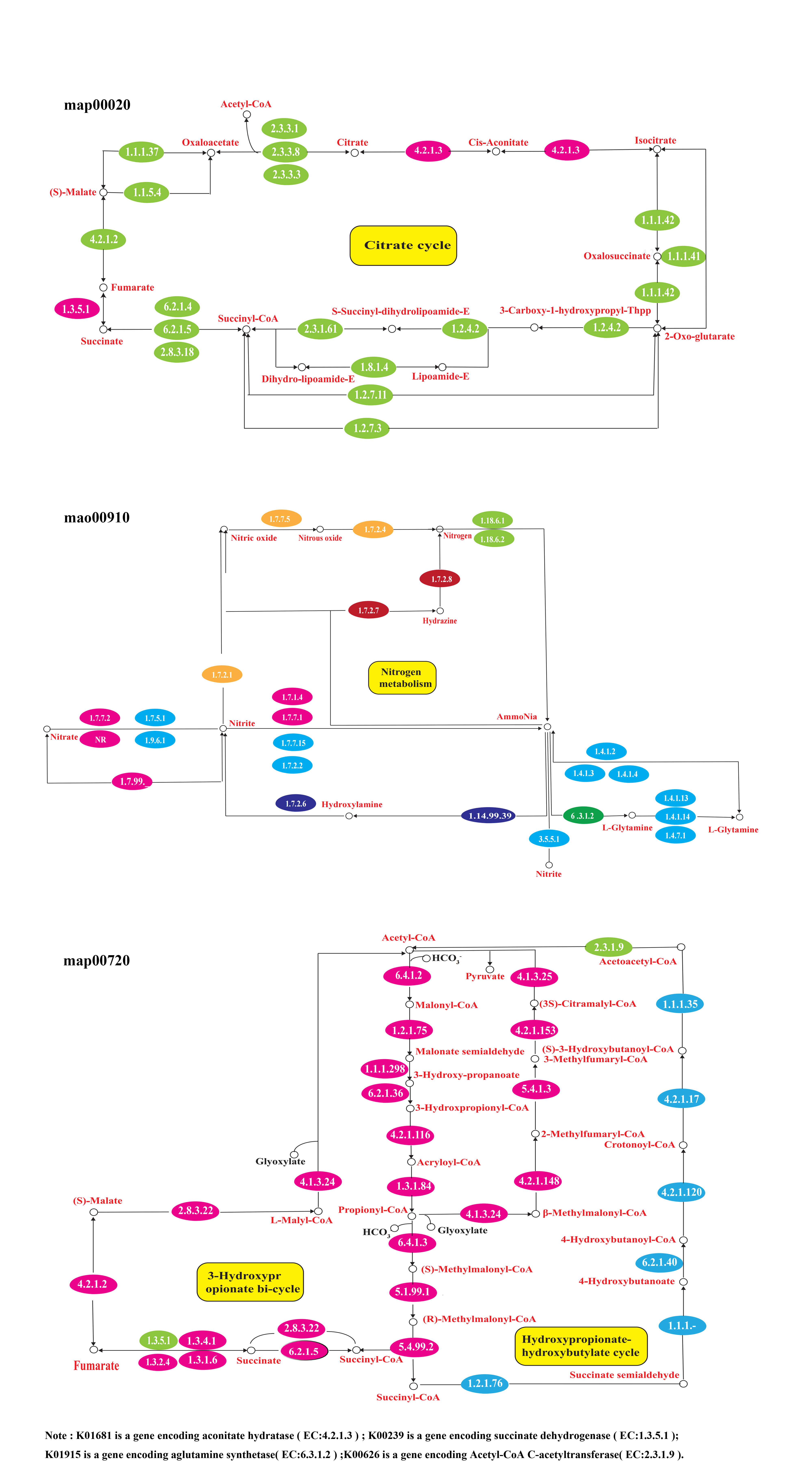

Supplement: Supplementary file 5 [file Image_1.png]
